# A Workout to Remember: CrossFit-Induced Vertebral Artery Dissection

**DOI:** 10.7759/cureus.41211

**Published:** 2023-06-30

**Authors:** Kyle C Buckley, Amanda Luu, Janet Grotticelli, Sonu Sahni

**Affiliations:** 1 Primary Care, Touro College of Osteopathic Medicine, New York, USA; 2 Pulmonary Medicine, Harlem Hospital Center, New York, USA; 3 Research Medicine, New York Institute of Technology College of Osteopathic Medicine, New York, USA

**Keywords:** horner’s syndrome, stroke, cerebellar infarct, cervical manipulation, crossfit, cranial nerve palsy, cerebrovascular accident, vertebral artery dissection

## Abstract

Vertebral artery dissection (VAD) is a cause of brain stem stroke in the younger population and is commonly associated with trauma, sudden neck movement, or chiropractic manipulations. In this case, a 28-year-old male with a non-significant past medical history who recently started a CrossFit boot camp presented to the emergency department with right-sided neck pain, dysarthria, mild right gaze paresis, right dysmetria, and right facial droop with symptoms of Horner’s syndrome. Imaging results revealed an age-indeterminate left lateral cerebellar infarct with right VAD. The goal of management is to prevent stroke, which is done with anticoagulation and potentially thrombolytic therapy if there are no contraindications. The patient received thrombolytic therapy and was treated with conservative management. The prognosis is good for patients who survive the initial dissection and are treated in this manner. It is important to obtain a thorough history of young and healthy patients who present with concerning neurologic symptoms so that precipitating activities are not missed.

## Introduction

Vertebral artery dissection (VAD) is a common cause of cerebrovascular accidents in the younger population (ages 18-45) with an estimated incidence of approximately 1-1.5 per 100,000 individuals [1]. Possible risk factors for VAD include connective tissue disorders, hyperhomocysteinemia, recent infection, α1-antitrypsin deficiency, and a variety of neck movements [2]. The presenting signs and symptoms may be vague, and the diagnosis can be delayed. Initially, patients typically complain of unilateral neck pain and/or headache in the occipitocervical area [3]. Often neurological symptoms do not occur or present late. Yet, 70% of patients have some type of neurological deficit [3]. This report presents a case of VAD in a young patient caused by strenuous CrossFit training. Our aim is to underline CrossFit training as a risk factor for VAD and emphasize a low threshold of suspicion when evaluating these patients.

This case report was previously presented as an abstract and poster submission at the American Thoracic Society International Conference on May 22, 2019.

## Case presentation

**Figure 1:**
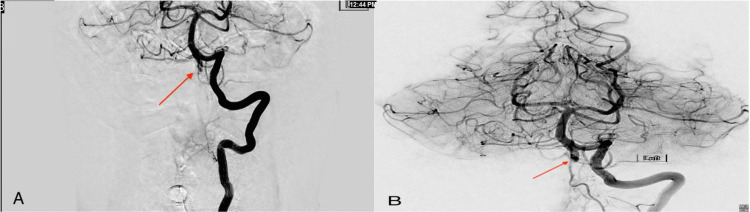
Computed tomography angiography. Patent vertebrobasilar junction and basilar apex with no evidence of large-vessel occlusion. There was a spontaneously recanalized distal right posterior cerebral artery occlusion and right cervical vertebral artery dissection.

**Figure 2:**
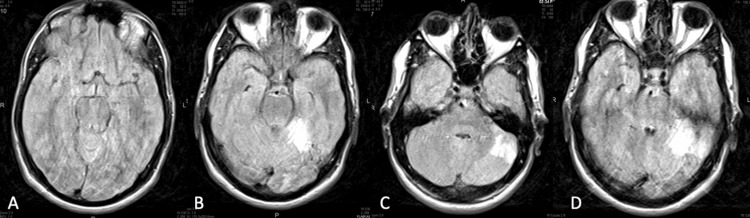
Magnetic resonance imaging/Magnetic resonance angiography. Bilateral inferior cerebellar hemisphere (posterior inferior cerebellar artery territories) patchy acute and subacute infarctions, possible right medulla subacute infarction, left superolateral cerebellar hemisphere subacute infarction, right thalamus medial focal acute infarction, and absent right vertebral artery flow void, suggesting either occlusion or reduced flow.

A 28-year-old male with a non-significant past medical history who recently started a CrossFit boot camp began to complain of sudden-onset, right-sided neck pain radiating to the right frontal region, confusion, bilateral arm numbness, and ataxia. He denied any trauma or activities involving sudden neck movements. The patient was seen in an urgent care center and was found to be alert, awake, and oriented without any neurological deficits on examination, and was discharged home without any imaging. Over the following two weeks, his symptoms worsened, prompting a visit to the emergency department. On presentation, the patient complained of right-sided neck pain and was found to have dysarthria, mild right gaze paresis, right dysmetria, and right facial droop with symptoms of Horner’s syndrome. The patient’s National Institute of Health Stroke Scale (NIHSS) score was 6, resulting in the initiation of a stroke code. A computed tomography (CT) scan of the head revealed an age-indeterminate left lateral cerebellar infarct. A CT angiography study (Figure 1) showed possible right VAD. Magnetic resonance imaging/Magnetic resonance angiography studies (Figure 2) were subsequently performed which displayed multiple areas of infarction, as well as either slow or potentially absent blood flow through the right vertebral artery. The patient received thrombolytic therapy with tissue plasminogen activator, and thereafter his neurological status deteriorated with an NIHSS of 16. The patient was intubated and a repeat CT scan was negative for acute bleeding. Further angiography studies confirmed right VAD starting at the origin (V1) and extending up to C2 (V3), in addition to an occlusion of the right posterior inferior cerebellar artery. Subsequently, the patient’s symptoms eventually improved, and he was extubated and discharged to outpatient rehab. The patient is currently doing well with minimal residual neurological defects.

## Discussion

Incidence

VAD is responsible for approximately 20% of ischemic strokes in young people and can be precipitated by connective tissue disorders, hyperhomocysteinemia, recent infection, α1-antitrypsin deficiency, and a variety of neck movements [2,3]. In this report, a case involving the association between CrossFit and a spontaneous VAD is discussed. In a review of the existing literature, Lu et al. presented three separate case reports of CrossFit-related cervical internal carotid artery dissection, with consequences ranging from partial Horner’s syndrome to extensive cerebral infarcts [4]. Carotid artery dissections are three to five times more common than VADs [3]. This is possibly due to the protective course of segment two of the vertebral artery through the cervical transverse foramina extending upward from C6 to C2 [3]. Segment three is the tortuous segment that starts at the transverse foramina of C2 and runs in the posterolateral part, looping around C1 and then passing between the atlas and the occiput. The majority of spontaneous dissections occur in segment three, where the vertebral artery has exited the transverse foramina [3].

History

According to Rodriguez et al., there is a prevalence of VAD in certain high-intensity sports that include but are not limited to mixed martial arts, kickboxing, rugby, weightlifting, and gymnastics [5]. A CrossFit workout consists of constantly varied functional movements performed at high intensity that are designed to improve fitness and health. A workout of the day (WOD) combines traditional cardiovascular exercises, such as running, biking, and rowing, with elements from Olympic weightlifting, powerlifting, strongman, and gymnastics [6]. Thus, an overlap can be seen between sports that have been shown to cause VAD and the fundamental basis of a CrossFit WOD. In this light, participation in CrossFit may be a risk factor for spontaneous VAD and must be considered by clinicians while obtaining a patient’s history.

Symptomatology

In addition to a thorough history, patient symptomatology must be scrutinized. According to the systematic review conducted by Gottesman et al., data suggest that VAD may have a higher prevalence, even among patients with non-specific, mild symptoms such as headache or dizziness. The symptomatology of VAD has been outlined in Table 1.

**Table 1:** Symptomatology of vertebral artery dissection. Table referenced from Gottesman et al. (2012) [1]. Permission to reproduce the table was obtained from Wolters Kluwer Health, Inc.

Symptom	Total sample size (N)	Number of subjects with symptom
Dizziness/Vertigo	467	273
Headache	689	348
Neck pain	526	244
Gait problems/Ataxia	150	57
Visual symptoms	314	114
Nausea/Vomiting	306	108
Nystagmus	150	44
Horner’s syndrome	265	58
Sensory deficits	335	70
Cranial nerve palsies	241	51
Dysphagia	102	13
Tinnitus	238	17

The patient in this report initially complained of right unilateral neck pain with radiation to the right frontal region, bilateral arm numbness, ataxia, and confusion. However, the neurological symptoms occurred before the urgent care center visit and appeared to be transient, which delayed further testing and treatment. Upon arrival at the emergency department two weeks later, the patient presented with significant right-sided neurological deficits, which is consistent with the literature regarding their initial transient nature and resultant late onset [3]. Therefore, it is suggested that VAD should be considered in the differential diagnosis in patients with common symptoms, even in the absence of hemiparesis or cranial nerve palsy [1]. Given his participation in CrossFit, along with his early symptomatology, VAD should be listed as a differential diagnosis for this patient and ruled out clinically.

Conservative management and neurointervention

The goal of conservative management of VAD is to prevent stroke and long-term neurological complications [3]. Patients are typically started on anticoagulation such as heparin, and if there are no contraindications, and it is within 4.5 hours from the onset of symptoms, the patient may also be given antithrombotics [3]. Hospital admission is required, along with strict monitoring of neurological symptoms during the course of management. Of the patients treated in this manner, approximately 80% achieve a full recovery [3].

Candidates for neurointervention include patients with recurrent ischemia despite medical treatment, patients with contraindications to anticoagulants or antiplatelet medications, and patients with significantly compromised cerebral blood flow [7]. These patients are treated with angioplasty, stent, or stent-supported angioplasty, whereby a metal cage is deployed within the artery, which acts as a scaffold that is used to tack down the intimal flap and reinforce the vertebral artery wall [7]. Postulated benefits of such an intervention are enhanced arterial wall strength, prevention of recurrence, more rapid resolution of symptoms, and prevention of pseudoaneurysm formation [7]. However, the role of this therapy remains controversial as most patients can be managed successfully with anticoagulation therapy, and most dissections repair on their own [3].

Sequelae and prognosis

Complications of VAD include cerebellar and brain stem infarction, subarachnoid hemorrhage, and vertebral artery pseudoaneurysm leading to compression neuropathy of the cranial nerves [3]. For patients who survive the initial extracranial dissection, the prognosis is good with complete recovery in 80-90% of individuals [3]. However, at least 10% will develop recurrent dissection, a major stroke, or death [3]. Patients who have severe neurological deficits at the time of presentation, or who develop an intracranial dissection, have a poor prognosis [3]. Intracranial dissections are often associated with brain stem infarctions, subarachnoid hemorrhage, and death [3].

## Conclusions

As presented in this case, it is possible for CrossFit workouts to be so strenuous that they can induce VAD in relatively healthy young adults. It is important to obtain a thorough history of young and healthy patients who present with concerning symptoms so that precipitating activities are not missed. Further studies of cases are needed to determine the likelihood of which CrossFit activities can cause VAD and how to potentially prevent VAD from occurring as a result of exercise.
